# Electrochemical Measures for Determining the Total Antioxidant Capacity of Açaí Pulp (*Euterpe oleracea*) at a Glassy Carbon Electrode

**DOI:** 10.3390/antiox14091082

**Published:** 2025-09-03

**Authors:** Tabata N. Feijoó, Luis D. Loor-Urgilés, Danyelle M. de Araújo, Elisama V. dos Santos, Marília Oliveira Fonseca Goulart, Carlos A. Martínez-Huitle

**Affiliations:** 1 Renewable Energies and Environmental Sustainability Research Group, Institute of Chemistry, Federal University of Rio Grande do Norte, Lagoa Nova, Natal CEP 59078-970, RN, Brazil; tabata.feijoo.721@ufrn.edu.br (T.N.F.); luis.loor.172@ufrn.edu.br (L.D.L.-U.); danyelle.medeiros@ifrn.edu.br (D.M.d.A.); elisama.vieira@ufrn.br (E.V.d.S.); 2 Instituto de Química e Biotecnologia, Universidade Federal de Alagoas, Av. Lourival Melo Mota s/n, Campus Universitário, Tabuleiro do Martins, Maceio CEP 57072-970, AL, Brazil; mofg@qui.ufal.br

**Keywords:** electrochemical index, total antioxidant power, açaí, glassy carbon

## Abstract

Antioxidants, such as flavonoids, are influential secondary metabolites that play a significant role in regulating human health. Açaí, known for its potent antioxidant properties, has gained popularity in the nutritional field. However, there is a need for accurate methods to quantify its antioxidant capacity. Therefore, the goal of this investigation was to determine the total antioxidant capacity of frozen açaí pulp by applying the concept of the electrochemical quantitative index (EQI) using the cyclic voltammetry technique. The electrochemical response of ethanolic extracts obtained by a nonconventional ultrasound bath was investigated in the anodic region. The results clearly showed redox behavior at +0.37 V and +0.27 V (vs. Ag/AgCl) for the anodic and cathodic peaks, respectively, when evaluated by cyclic voltammetry at a glassy carbon (GC) electrode. By investigating a constant ethanolic extract concentration (0.2%) and analyzing the scan rate and supporting electrolyte effects, it was determined that the frozen açaí pulp extract presented an EQI of about 2.3 µA/V. Similarly, the concept of the EQI was extended to the use of the differential pulse voltammetry profile of a 0.2% ethanolic açaí extract on different supporting electrolytes, which showed that some experimental conditions needed improvement. Still, maintaining pH with a buffer solution in the anodic region is crucial to ensure reproducibility. The antioxidant capacity was also determined using the 2,2-diphenyl-1-picrylhydrazyl (DPPH) free radical assay to compare the electrochemical results. The Folin–Ciocalteu colorimetric test was applied to determine the total phenolic content of the extract.

## 1. Introduction

**Figure 1:**
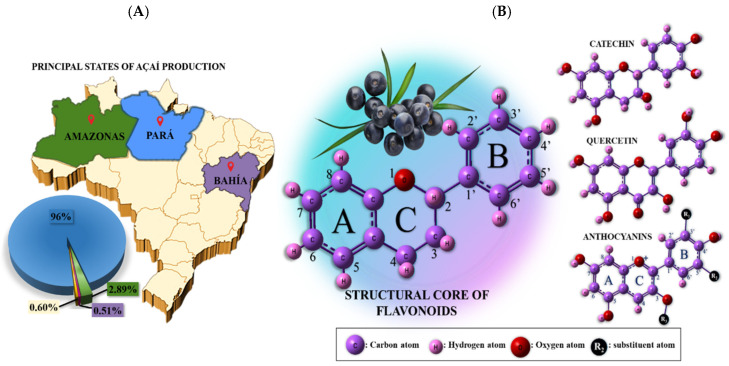
(**A**) Map of Brazil highlighting the central regions of açaí production. (**B**) Açaí fruit and its main flavonoid’s components.

The berry fruit açaí is touted as a “superfruit” due to its high content of antioxidants in both the pulp and the seed. Depending on the area and cultural setting, this plant is referred to as acai, huasaí, or naidí [1,2,3], and it grows mainly along the Amazon River in the states of Pará, Amazonas, and Bahia (Figure 1A), providing a significant economic impact on the rainforest region due to its high demand throughout the country and the whole world. However, around 85% of the fruit is discarded after pulping, as only 15% of the fruit corresponds to the pulp and is utilized for human consumption in the form of juice, energy drinks, açaí bowls, ice creams, and other products. Due to açaí’s high popularity and consumption, roughly 1.7 million tons of berries was collected in 2022, of which 8200 tons was destined for exportation and the rest for local consumption, which means that Brazil generated close to USD 1.16 million from the annual production of açaí alone [2,4]. During this industrial process, significant amounts of commercial products and residues are produced. Then, the identification of new chemical features and applications of açaí, as well as the recovery of nutrients from its residues and their valorization, are promising approaches to promote the transition from the current economy to a circular one.

In general, the health-promoting antioxidant impact of red fruits [5,6,7] like açaí is attributed to their rich content of flavonoids such as catechin, quercetin, and anthocyanins (Figure 1B), which are highly conjugated phenolic compounds produced biosynthetically from phenylalanine. These bioactive compounds, or low-molecular-weight secondary metabolites, primarily act as reducing agents against radical species due to their structural functionality, which enables their stability and shields cells from oxidative damage [8]. There are other indirect mechanistic possibilities for their action as antioxidants, such as the chelation of metallic ions, which limits the availability of metal ions that catalyze harmful reactions [9,10,11,12]. This characteristic is attributed to the oxidation of hydroxyl groups, primarily in the B ring of the flavonoid nucleus (Figure 1B); thus, the number and molecular position of -OH groups influence the antioxidant capacity of samples [13]. The succeeding antioxidant free radical breaks the chain reaction and stops the creation of additional free radicals due to the stability brought about by the flavonoids/other phytochemicals’ radical delocalization.

In this context, investigating the antioxidant potential in natural food matrices is highly relevant, particularly when considering the growing interest in functional foods and clean-label products. Recently, various electrochemical techniques have garnered increased attention as tools for in vitro and in situ investigations related to the determination of the total antioxidant capacity of several fruits, foods, and plant extracts. The application of electrochemical methodologies is made possible by the ability of electrode materials or oxidant compounds to donate or receive electrons [14,15]. Nevertheless, the evaluation of an antioxidant’s capacity by electrochemical methods is often achieved by collecting information regarding its performance in a specific environment, even though its activity involves several mechanisms that do not depend on a single direct process [16,17]. Therefore, electrochemical assays are not universally accepted as reliable techniques, even when the results reported in the existing literature are comparable to those obtained using traditional methods to determine antioxidant activity. Indeed, there is no widely adopted “total antioxidant parameter” as a nutritional index for labeling food and biological fluids. Concerning electron transfer (ET)-based methods for analytical, food chemical, biomedical/clinical, and environmental scientific communities, they can be effectively used to meet specific communities’ needs.

Within this framework, different studies have demonstrated that the electrochemical quantitative index (EQI), mainly achieved by cyclic voltammetry, can be correlated with total antioxidant capacity in ET-based processes, with a confidence [18,19,20,21,22] similar to other traditional methods (spectrophotometric and chromatographic). Electroanalytical approaches can be considered fast methods and hold certain advantages over conventional ones; for instance, they can be selective depending on the properties of the electrode used and the experimental conditions. In the case of electrode materials, they can achieve low detection limits by using small sample amounts [14,23,24], with highly reproducible responses, compactness, low cost, selectivity, sensitivity, portability, and multiplexed and in situ analysis for different inorganic/organic compounds. Nevertheless, some disadvantages can be considered, such as an inability to identify the structure of compounds, which is also a limitation of other methods. However, it is possible to integrate instrumental methods, such as liquid chromatography combined with mass spectrometry (LC-MS). With these hybrid techniques, they become superior due to their lower detection limits and higher sensitivity and accuracy [25]. Despite this, electrochemical techniques are still beneficial in analytical chemistry due to their robustness, affordability, and adaptable nature, providing essential data where some optical and advanced methods may fall short. Therefore, further investigations should be conducted to introduce these strategies into antioxidant research.

Research has been extended to the applicability of other voltammetric analyses, such as differential pulse (DPV) and square wave (SWV) voltammetries, which are electrochemical techniques widely used to collect both the qualitative and quantitative profiles of redox compounds [26], including the mechanisms and kinetics of electron transfer reactions [27]. This information prompts us to explore the feasibility of determining the antioxidant capacity of an ethanolic extract from commercial açaí pulp based on the EQI by measuring the anodic current and potential in CV and DPV using a glassy carbon (GC) electrode.

The commercial açaí pulps used in this study correspond to minimally processed, frozen food-grade products intended for direct human consumption, representing complex natural matrices. To better understand their electrochemical behavior, we investigated the impact of extract concentrations at different scan rates and with various supporting electrolytes, including phosphate buffer solution, sodium chloride, and sodium nitrate, in natural complex samples. Finally, the results were compared with those obtained from the DPPH free radical assay and the Folin–Ciocalteu test to obtain the total phenolic content of the ethanolic extract.

The present study contributes to the growing body of knowledge on the electrochemical determination of antioxidants by exploring its application to commercial açaí pulps, providing insights into their redox behavior under various electrolytic conditions, and evaluating the technique’s analytical potential in natural food matrices.

## 2. Materials

### Chemical Reagents

Monobasic sodium phosphate NaH_2_PO_4_ (Sigma-Aldrich, São Paulo, Brazil), dibasic sodium phosphate Na_2_HPO_4_ (Dinâmica^®^ Ltda, São Paulo, Brazil), sodium chloride NaCl (Dinâmica^®^ Ltda, São Paulo, Brazil), sodium nitrate NaNO_3_ (Synth, São Paulo, Brazil), sodium carbonate Na_2_CO_3_ (Dinâmica^®^ Ltda, São Paulo, Brazil), gallic acid C_7_H_6_O_5_ (Sigma-Aldrich, São Paulo, Brazil), and ethanol EtOH (Atriom, São Paulo, Brazil) were used. All solutions were prepared with Milli-Q ultra-purified distilled water (resistivity ≥ 18.2 MΩ cm, Millipore at 25 °C) and used without further purification. The stable free radical 2,2-diphenyl-1-picrylhydrazyl (DPPH) was obtained from Sigma-Aldrich (São Paulo, Brazil), and the Folin–Ciocalteu reagent was obtained from Êxodo Científica (São Paulo, Brazil).

## 3. Methodology

### 3.1. Extraction and Sample Preparation

Commercially preserved açaí pulp (47.77 g), purchased from the brand Pé de Fruta^®^ (Paraíba, Brazil), was thawed at room temperature and extracted with 10 mL of EtOH 99% in an ultrasonic bath for 15 min, as already reported in the existing literature [21]. The crude material was then centrifuged (NT 810, Novatecnica, Piracicaba, Brazil) at 1000 rpm for 10 min and filtered through an 8 µm pore size filtration paper. No solid residue was obtained from the commercial açaí pulp, which was a fine powder. The sample was stored at −18 °C, after which a 100 µL aliquot was added to the supporting electrolytes, resulting in a total volume of 25 mL and a 0.2% extract concentration for all electrochemical assays. Extraction and sample preparation was performed 48 h before each experiment in the absence of light at room temperature (20 °C).

### 3.2. Total Phenolic Content (TPC)

The total phenolic content of the ethanolic extract was determined using the Folin–Ciocalteu colorimetric method, following the procedure described by Costa et al., 2024 [28], with slight modifications. Briefly, 0.1 mL of the ethanolic extract was mixed with 6 mL of ultrapure Milli-Q water and 0.5 mL of Folin–Ciocalteu reagent. After 8 min, 1.5 mL of 7.5% (*w*/*v*) Na_2_CO_3_ solution was added, and the volume was adjusted to 10 mL with ultrapure Milli-Q water. The mixture was kept in the dark at room temperature for 120 min. After the reaction was completed, the resulting solution was diluted 1:10 with ultrapure water, and absorbance was measured at 765 nm using a Thermo Fisher Scientific Model Genesys 180 spectrophotometer (Waltham, MA, USA). A calibration curve was constructed using gallic acid as the standard (0–100 mg mL^−1^), and the results are expressed as mg of gallic acid equivalents per mL of extract (mg GAE mL^−1^) and as mg GAE g^−1^ of fresh pulp.

### 3.3. DPPH Assay

The antioxidant capacity of açaí was determined by radical scavenging using the DPPH method. Aliquots of 0.30 mL of the extract dissolved in ethanol (0.2% of ethanolic extract) were mixed with 2.7 mL of DPPH solution (40 µg mL^−1^ in methanol) [29]. The mixture was then homogenized and stored in the dark for 30 min, and measurements were performed at 517 nm using a UV-Vis spectrophotometer (Shimadzu UV 1800, Berlin, Germany). The analyses were performed in triplicate (*n* = 3) and the percentage inhibition or the EC_50_ (half maximal inhibitory concentration, which represents antioxidant capacity or antioxidant power) was calculated graphically using an analytical curve in the linear range, plotting the extract concentration versus the corresponding scavenging effect Equation (1) at 30 min [29].

DPPH scavenger effect or EC_50_ (%) = [(A_0_ − A_1_)/A_0_] × 100 (1)

where A_0_ and A_1_ correspond to the absorbance at *λ* = 517 nm of the DPPH radical in the absence and the presence of the antioxidant, respectively [29].

### 3.4. Electrochemical Assays

All electrochemical measurements were performed in a single-compartment electrochemical cell using the potentiostat/galvanostat model PGSTAT302N. A GC electrode with a 3.0 mm diameter (area = 7.1 mm^2^) served as the working electrode, while Ag/AgCl (3 M KCl) and a platinum wire were used as the reference and counter electrodes, respectively. Different supporting electrolytes (SEs) were tested, such as NaCl (0.1 M) and NaNO_3_ (0.1 M), with the pH adjusted using a phosphate buffer solution (PBS, 0.1 M) and without pH adjustment, to avoid the decomposition of active compounds in the extract [21]. Based on the existing literature, most of the antioxidant compounds present in açaí extract are polyphenols, which are bioactive compounds that have oxidation potentials between 0 and +1.0 V (vs Ag/AgCl) [20].

The experimental conditions for cyclic voltammetry (CV) included a potential range from 0 to +0.90 V at a scan rate range from 60 to 240 mV s^−1^, while those for differential pulse voltammetry (DPV) used a modulation amplitude of 50 mV, a modulation time of 500 ms, step potential of 5 mV, and scan rate of 100 mV s^−1^. Before each measurement, the GC surface was polished with 0.05 µm alumina powder on a microcloth pad, rinsed thoroughly with distilled water, and then placed in an ultrasonic bath.

The total antioxidant power was evaluated by the determination of the EQI [18,19,20,21,22], estimated via the current responses of the cyclic voltammograms and Equation (2), where *I_ap_* and *E_ap_* are the anodic current intensity and the oxidation potential,

(2) $$EQI=\frac{I_{{ap}_{1}}}{E_{{ap}_{1}}}+\frac{I_{{ap}_{2}}}{E_{{ap}_{2}}}+\cdots +\frac{I_{{ap}_{n}}}{E_{{ap}_{n}}}$$

## 4. Results and Discussions

### 4.1. Concentration Test

**Figure 2:**
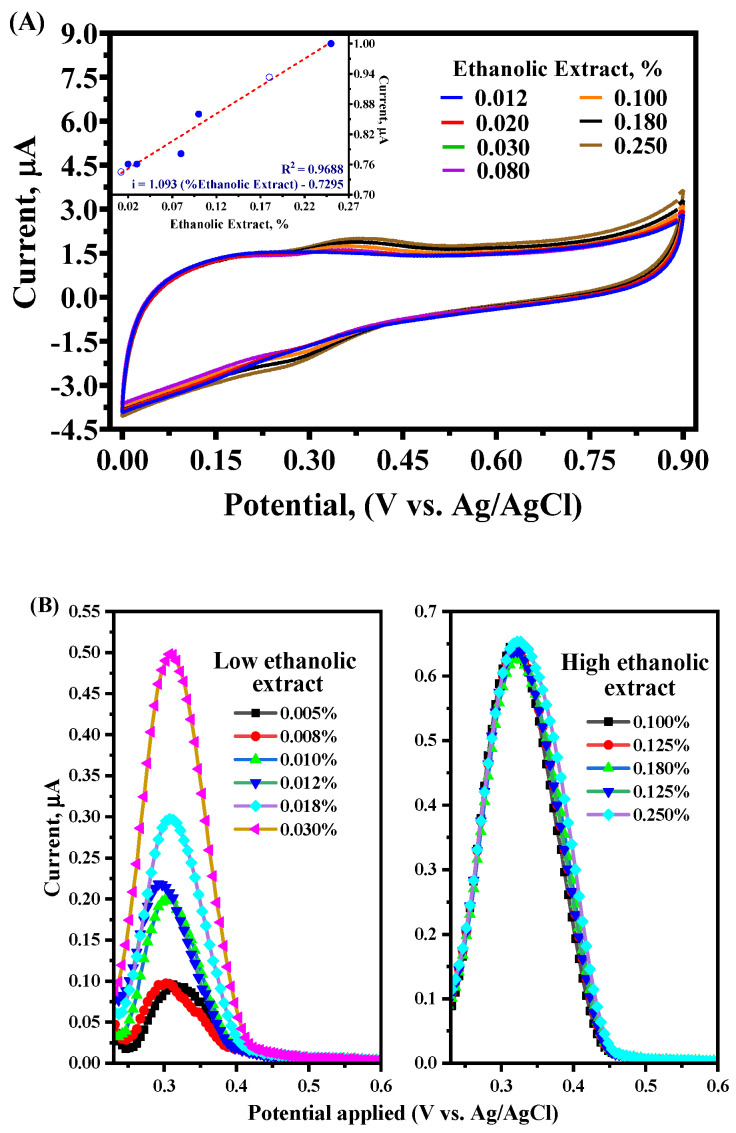

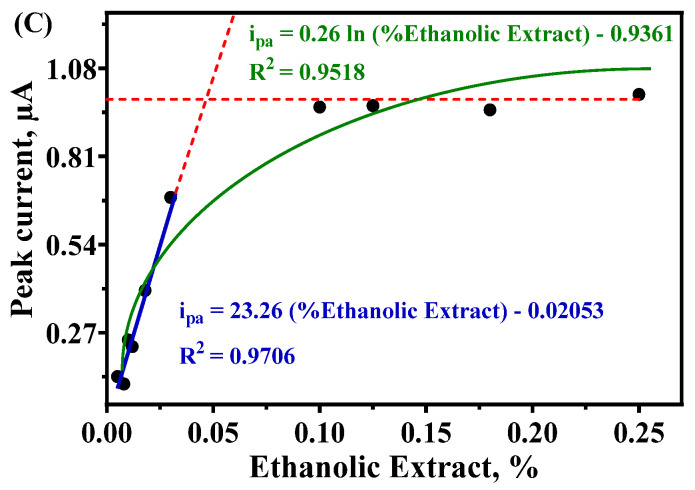
(**A**) CV and (**B**) DPV profiles at GC electrode in phosphate buffer solution (0.1 M) pH 5 with different concentrations of commercial açaí pulp extract at scan rate 100 mV s^−1^, the inset plot in CV measurements shows the analytical curve obtained. (**C**) Analytical curve of the anodic peak current obtained by DPV measurements at different ethanolic extract concentrations. In (**A**), the red dashed line represents the linear fit of the experimental data, whereas in (**C**), the red dashed lines indicate the two linear segments identified within the logarithmic curve.

To assess the total antioxidant power of the ethanolic extract, preliminary assays (CV and DPV measurements) were performed in a pH 5 phosphate buffer 0.1 M solution at a GC electrode to understand its electrochemical behavior. As shown in Figure 2A,B, specific electrochemical responses were achieved. In the case of CV, no current signals were registered in the voltammetric profile in the absence of açaí extract in the solution (black solid line in Figure 2A). Meanwhile, when different concentrations of açaí extract were added to the solution, it was possible to observe the presence of a pair of signals, anodic (+0.375 V) and cathodic (+0.280 V) peaks, respectively, related to the electroactive species. When a lower concentration of the ethanolic extract was used (0.012–0.030%), several cycles were registered and no passivation was observed, indicating that no adsorption of any compound was attained at the GC surface. Even when a slight increase in the current response was achieved at both signals with different concentrations of the extract, a good linear relationship of the peak current versus the concentration of açaí extract (0.012% to 0.25%) was revealed (see the inset in Figure 2A). This behavior can be attributed to the presence of antioxidant species in the solution containing redox active functional groups, increasing the current response with an increase in the concentration of the compounds [18,19,20,21,22]. These findings support that analytical curves can be built using information obtained by CV profiles [30,31], as well as the estimation of the EQI to determine antioxidant capacity, as already conducted by other authors [18,19,20,21,22]. Nevertheless, it is relevant to demonstrate if a linear trend is shown by a more accurate protocol, as well as to test other experimental conditions. Thus, the current profiles were acquired by DPV analysis to comprehend the selected range of açai concentrations.

DPV profiles were recorded under the experimental conditions described in Section 2, showing an increase in the current response with an increasing ethanolic extract concentration. However, two behaviors could be observed at different concentration ranges of the extract. On one hand, continuous current increases were attained at lower extract concentrations (Figure 2B). On the other hand, no significant current increases were observed at higher extract concentrations (Figure 2B). In fact, when all current data was plotted as a function of ethanolic extract concentrations, a logarithmic trend was obtained considering both behaviors described above (Figure 2C). Conversely, two linear segments could be associated according to the selected extract concentration (Figure 2C), obtaining the following different correlation coefficients: (i) at lower concentrations (0.005% to 0.03%), there was a value of about 0.97, following a linear relationship, while (ii) at higher concentrations (0.1% to 0.25%), the value of R^2^ was about 0.96, rather than following a linear relationship. In the former, a more accurate extract concentration range was achieved, resulting in a better sensitivity to lower antioxidant concentrations. Meanwhile, the saturation of the system and electrode surface could be associated with the higher concentrations used, leading to a plateau in the latter. This pattern resembles the characteristics of electrochemical systems, where the response at high concentrations may be limited in the case of saturation of an electrode’s active sites or charge transfer mechanisms [32]. Although the DPV signal appeared to be constant within the extract concentration range of 0.1–0.2%, this information helps to establish the maximum antioxidant capacity that can be measured using a more electrochemically accurate protocol, such as DPV, under specific experimental conditions.

### 4.2. Supporting Electrolyte Analysis

**Figure 3:**
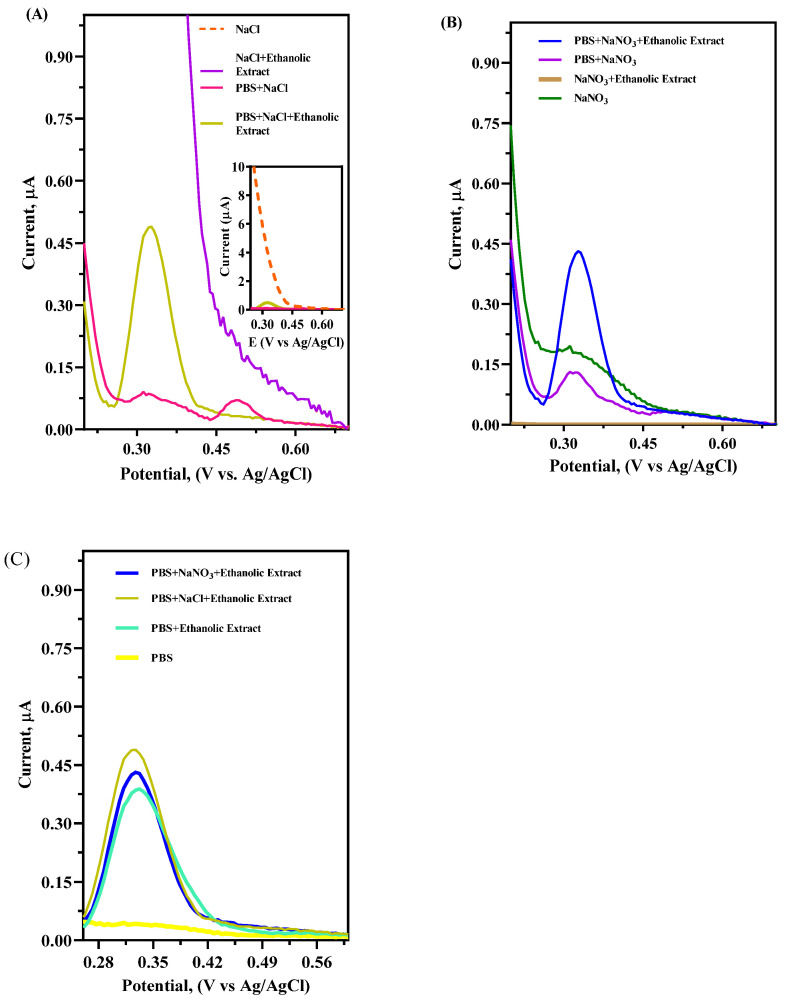
DVP profiles at different SE solutions with GC electrode adding 0.1 M phosphate buffer solution (pH 5) with açaí pulp extract (0.2%) at scan rate 100 mV s^−1^: (**A**) NaCl (0.1 M); (**B**) NaNO_3_ (0.1 M); and (**C**) comparative plots. The inset in Figure 3A corresponds to the zoom area for PBS + SE + Ethanolic extract profile.

The selection of the appropriate SE for determining the total antioxidant capacity is relevant to facilitate electron transport by modifying the electric field at the electrical double layer [33]. Therefore, the voltammetric response was registered by testing different SEs, such as 0.1 M NaCl and 0.1 M NaNO_3_, adjusting the pH with 0.1 M phosphate buffer (pH 5) solution (Figure 3) to avoid the decomposition of active compounds in the extract [21]. A potential window for the electrochemical measurements between 0 and +1.0 V (vs Ag/AgCl) [20] was selected, once the bioactive compounds in açaí had oxidation potentials in that range.

Based on the results obtained in all SE solutions, the anodic [22,33,34,35] signal at about +0.33 V (vs. Ag/AgCl) could be attributed to the antioxidants in the sample, as it only appeared when 0.1 mL of the ethanolic extract was added to the solution. Upon performing DPV on açaí extract with PBS, NaCl, NaNO_3_, PBS + NaCl, and PBS + NaNO_3_, analogous behavior was observed in all solutions. As shown in Figure 3, the DPV profiles exhibited significant changes depending on the SE used and the adjustment of pH conditions with the PBS solution. The pH values influenced the redox activity of the bioactive compounds at the GC surface. The presence of NaCl enabled achieving a higher peak current response (Figure 3C), and, consequently, it was selected as the SE for the subsequent experiments [21]. Considering this, the use of a strong electrolyte in combination with a buffer solution can be regarded as an essential tool to ensure the reproducibility of the method [15].

### 4.3. Scan Rate Test

**Figure 4:**
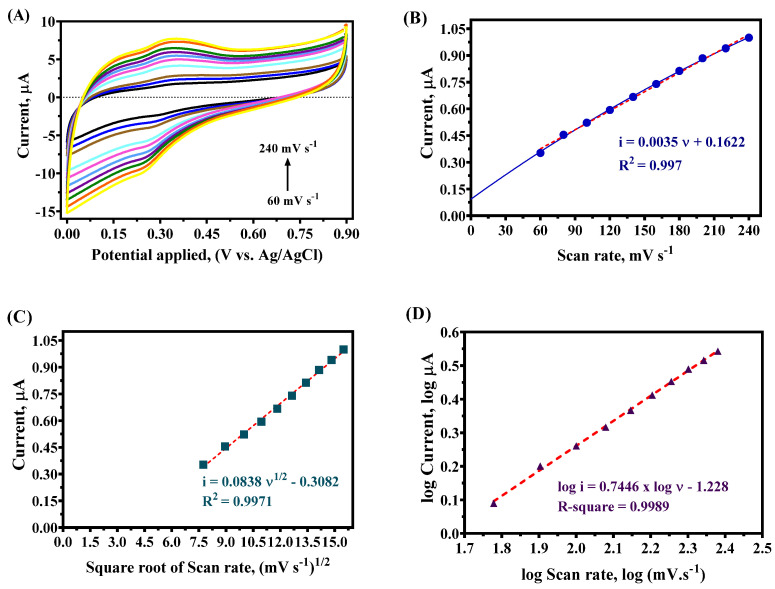
(**A**) CV profiles of ethanolic extract of açaí (0.2%) at different scan rates from 60 to 240 mV s^−1^ in PBS with NaCl using GC electrode. (**B**) Scan rate plot as a function of the current peak, (**C**) square root of scan rate plot as a function of the current peak, and (**D**) logarithm of scan rate plot as a function of the logarithm of the current peak in PBS with NaCl and ethanolic extract of açaí (0.2%). The red dashed line in (**B**–**D**) represent the linear fit of the experimental data.

The voltametric profiles of the açaí ethanolic extract in NaCl (pH 5) were registered to investigate the effect of the oxidation and reduction current–potential responses at different scan rates (60 to 240 mV s^−1^). Figure 4A depicts the oxidative and reductive signals as a function of the scan rate. These findings, also reported in Table 1, clearly reveal a linear correlation between the anodic current and the scan rate (Figure 4B), with a slope of 0.004. However, a slight nonlinear tendency (blue line in Figure 4A) indicates that the process obeyed combined mass transport conditions. Conversely, a stronger linear correlation (0.99) was observed when the anodic current was plotted as a function of the square root of the scan rate (Figure 4C), indicating that the mass transport process could also be driven by diffusion. Following the analysis of the linear relationship between log *i*_ap_ and log *n* (r^2^ = 0.99), which exhibited a slope of 0.75, it was determined that a combination of transport mechanisms governed the oxidation process of the extract on the GC surface. This slope, falling between 0.50 (indicative of diffusion control) and 1.0 (indicative of adsorption control), suggests a dual influence of mass transport on the process [35]. Additionally, the study related to the current response at different scan rates with an açaí extract concentration of about 0.2% was consistent and predictable.

### 4.4. Antioxidant Power

**Figure 5:**
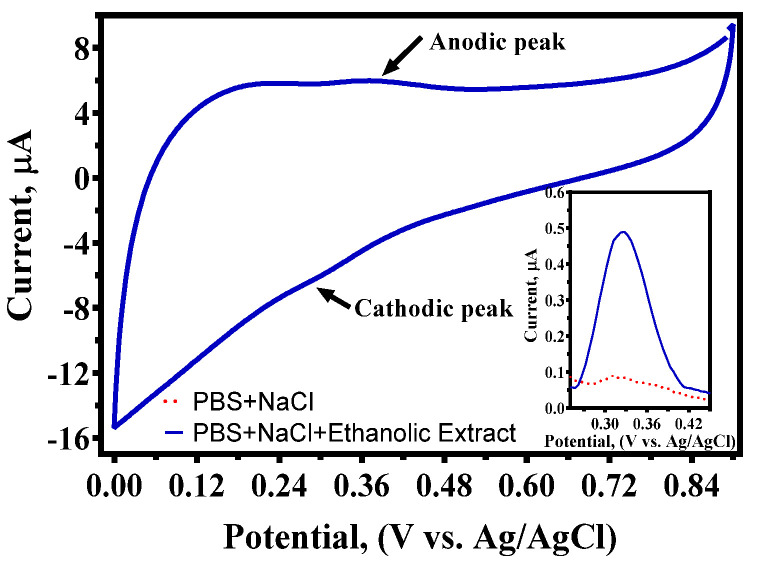
Cyclic voltammogram at GC electrode with açaí extract (0.2%) in phosphate buffer solution (0.1 M), pH 5, and sodium chloride (0.1 M) at scan rate 100 mV s^−1^. The inset graph shows differential pulse voltammograms under the same conditions as CV.

With the GC electrode polarized at a constant scan rate of 100 mV s^−1^, the Faradaic current recorded throughout the oxidation of the sample was used in a simple CV test to determine the antioxidant capacity of the açaí ethanolic extract based on the EQI. In a potential range from 0 to +0.9 V (vs Ag/AgCl), anodic and cathodic peaks appeared at +0.37 and +0.27 V, respectively, with E_1/2_ of +0.32 V, corresponding to the quasi-irreversible behavior of some antioxidant compounds present in the sample, as already confirmed by the CV profile when a commercial açaí extract was used (Figure 5). From these results, the EQI value for our ethanolic extract was determined by CV, which was about 2.3 ± 0.90 µA/V.

Meanwhile, the resolution of the oxidation peak was improved by correcting the DPV profile plotted in the inset of Figure 5, allowing us to estimate the EQI value in this work, which was approximately 1.08 ± 0.98 µA/V. Nevertheless, this value is different from data reported in the existing literature (see Table 2). These values were obtained with extracts from other red fruits, but with different protocols. Thus, these differences could be a result of various factors, such as the type of product used, chemical constituents of the sample, sample pre-treatment, interferences, amount of water, and concentration of phenolic compounds, as well as the electrochemical reactions, kinetic effects, and electrochemical protocols used to determine EQI values. For example, lower EQI values were achieved for *Rubus* spp. and *R. glabratus* Kunth. Conversely, higher EQI values were reported for blackberry jam fruit and acerola. In any case, the values obtained in our study indicate that the ethanolic extract of açaí has a moderate antioxidant capacity when compared with extracts obtained from other red fruits, as shown in Table 2. Within this framework, CV and DPV parameters could be used to estimate the EQI and, consequently, the antioxidant capacity of the açaí ethanolic extract.

### 4.5. Total Phenolic Content (TPC)

The TPC of the ethanolic extract of commercial açaí pulp was determined using the calibration equation y = 0.001 × −0.003 (R^2^ = 0.99), resulting in a value of 0.87 ± 0.01 mg GAE mL^−1^ of extract or 0.31 ± 0.002 mg GAE g^−1^ of fresh pulp (wet basis), after considering the dilution factor. This result is consistent with values previously reported for commercial açaí products. For instance, Belda-Galbis and co-workers [37] reported TPC values ranging from 0.70 to 2.1 mg GAE mL^−1^ in commercial açaí extracts, depending on the concentration. Conversely, Junior et al. [38] lyophilized açaí pulp and obtained a higher TPC value (0.77 mg GAE g^−1^), while Paz et al. [39] reported 18.08 mg GAE g^−1^ on a dry weight basis. These variations reflect differences in sample preparation, extraction method, and moisture content. Within this framework, TPC values are associated with the concentration of phenolic compounds capable of undergoing redox reactions with the Folin–Ciocalteu reagent, including anthocyanins and phenolic acids [28]. Although the method does not allow for the identification of specific compounds, previous studies have extensively described the phenolic composition of *Euterpe oleracea* using chromatographic techniques. The most frequently reported constituents include anthocyanins (e.g., cyanidin-3-glucoside, cyanidin-3-rutinoside, peonidin-3-glucoside, and peonidin-3-rutinoside) [40,41,42,43,44], phenolic acids (e.g., gallic, ferulic, vanillic, protocatechuic, syringic, and ellagic acids) [41,42,44], and other flavonoids, such as quercetin, catechin, rutin, epicatechin, and orientin [21,42,44]. These findings support the assertion that the ethanolic extract retained a significant load of bioactive phenolics, even after commercial processing.

### 4.6. DPPH Test

A spectrometric assay for radical scavenging activity was also conducted using the same samples (0.2% of ethanolic extract). In this method, the degree of DPPH radical scavenging, resulting from its reduction, indicates the antioxidant capacity. Therefore, the DPPH assay, expressed as EC_50_ (as described in Section 3.3), demonstrates a direct relationship with antioxidant activity. The EC_50_ value (in mg mL^−1^) obtained for the sample was 0.31 ± 0.15. This represents a significant total antioxidant concentration, which is related to the total phenolic concentration, which is directly proportional to the EQI, as previously reported in the literature [29]. The extract contained other chemicals besides flavonoids, including alkaloids, proteins, tannins, steroids, phenols, carbohydrates, and terpenoids, which affect the evaluation of the total phenolic concentration. Thus, the value of EC_50_ can be affected by the complex composition of the extract.

### 4.7. Assessment of Antioxidant Activity: Methods, Merits, and Limitations

It is worth noting that even though the EC_50_ is a standardized method accepted by the scientific community, different values of EC_50_ have been obtained for other fruits and vegetables, resulting in variations in its direct or indirect relationship with the EQI. Based on the existing literature [45], various techniques have been developed to quantify antioxidant activity (see Table 3), each with distinct mechanisms, advantages, and drawbacks. The most employed methods include DPPH, 2,2′-Azino-bis(3-ethylbenzothiazoline-6-sulfonic acid) (ABTS), ferric reducing antioxidant power (FRAP), oxygen radical absorbance capacity (ORAC), cupric reducing antioxidant capacity (CUPRAC), and TPC assays. Understanding the scope and limitations of these methods is crucial for selecting appropriate assays and interpreting results in both research and industrial settings [45].

Then, according to the information in Table 3, the diversity of antioxidant assays available today underscores the complexity of oxidative chemistry and the challenge of measuring the total antioxidant potential of food and pharmaceutical substances. Each method brings unique advantages tailored for specific sample types and chemical properties, but also carries inherent limitations that restrict universal applicability. For screening purposes, simple assays like DPPH and ABTS are practical. Meanwhile, for more biologically relevant assessments, ORAC offers a better physiological relevance, as indicated in Table 3. Conversely, FRAP and CUPRAC are excellent for reducing capacity quantification, while TPC provides a useful proxy for phenolic-rich matrices [45].

Within this framework, no single method is sufficient to characterize antioxidant activity comprehensively. Thus, a multi-method approach combining radical scavenging, reducing power, and content assays could be practical. Additionally, integrating cell-based and in vivo studies with these methods can provide a more accurate picture of antioxidant efficacy in real biological contexts. By comparing and contrasting these methods with the EQI approach, which is an emerging tool in antioxidant research, as proposed in this investigation, it is clearer that each of them could be used in different analyses. Comparison among them may be unfeasible due to their different mechanisms.

While conventional antioxidant assays measure total antioxidant capacity through radical scavenging or reducing power (see Table 4), the EQI provides molecular-level insight into the redox behavior of antioxidants [45]. For these reasons, these techniques are not mutually exclusive, but complementary. For example, for initial screening, assays like the EQI (by using CV), DPPH, and ABTS are sufficient due to their simplicity and low cost [45]. Meanwhile, the EQI, by DPV analysis, as a moderate or detailed characterization strategy, adds value by elucidating which groups are responsible for antioxidant behavior and their relative potency and ease of oxidation [45]. For regulatory or industry settings, standardized methods like FRAP and TPC are easier to implement, but the EQI (CV or DPV protocols) is gaining attention for its discriminatory power and real-time capabilities.

In studies where multi-component systems (e.g., botanical extracts and complex pharmaceuticals) are involved, combining spectrophotometric and electrochemical assays ensures both comprehensive quantification and compound-specific insights [45]. In this frame, it is possible to conclude that in our investigation, a direct comparison of EC_50_ values with the EQI could mask reliable information about antioxidant properties. Consequently, the EQI could be correlated with EC_50_ (as discussed in this work) and other standard antioxidant protocols under the same conditions to obtain a more accurate relationship between the antioxidant power of different samples. Therefore, more studies are needed to use electrochemical analysis for quality assessment and traceability of açai, as well as a detailed characterization protocol.

## 5. Conclusions

The use of electroanalytical assays (DPV and CV) enabled the quantification of the antioxidant capacity, as measured by the EQI, in a sample of frozen açaí pulp. Furthermore, this is expected to be reproducible and feasible for determining the antioxidant power in plant matrices using a GC electrode in a pH 5 PBS solution with NaCl as the supporting electrolyte. The total antioxidant capacity of the extract was quantified using voltammetric approaches and the DPPH spectrophotometric methodology, obtaining feasible results. Additionally, the Folin–Ciocalteu colorimetric method was applied to determine the total phenolic content, confirming the presence of redox-active phenolic compounds in the extract. This study confirms the strong antioxidant capacity of açaí pulp and that electrochemical assays are a functional, eco-friendly alternative method for quickly detecting antioxidants in food samples. Finally, the EQI represents a mechanistically grounded tool for assessing antioxidant capacity, offering distinct advantages in terms of sensitivity, specificity, and redox profiling. However, due to equipment and expertise requirements, it is not yet a full replacement for simpler and more widely adopted assays such as DPPH, ABTS, and FRAP. In future research, the integration of the EQI with conventional methods is likely to become standard practice, ensuring more reliable and mechanistically meaningful antioxidant evaluations, as performed in this work.

## Figures and Tables

**Table 1:** Values obtained from the investigation of the effect of the current/potential signals (Figure 4) at 0.2% concentration of the ethanolic extract of açaí as a function of scan rate at GC electrode (7.2 mm^2^).

*ѵ*, mV s^−1^	*E*_ap_, V	*E*_cp_, V	∆*E* = |*E*_cp_ − *E*_ap_|	*i*_ap_, µA	*i*_cp_, µA	∆*i* = |*i*_cp_ − *i*_ap_|
60	0.361	0.302	0.059	0.957	−1.131	2.088
80	0.368	0.292	0.076	1.301	−1.498	2.799
100	0.370	0.287	0.083	1.586	−1.837	3.423
120	0.373	0.283	0.090	1.976	−2.202	4.178
140	0.380	0.261	0.120	2.291	−2.743	5.033
160	0.383	0.258	0.125	2.572	−3.014	5.586
180	0.385	0.248	0.137	2.825	−3.472	6.298
200	0.392	0.246	0.146	3.119	−3.781	6.901
220	0.400	0.246	0.154	3.405	−4.056	7.461
240	0.402	0.239	0.164	3.647	−4.474	8.122

*i*_ap_ and *i*_cp_ are the anodic and cathodic current peaks, *E*_ap_ and *E*_cp_ are the anodic and cathodic potential peaks, and ***ѵ*** is the scan rate.

**Table 2:** Electrochemical quantitative index (EQI) obtained for different fruits with a GC as a working electrode.

Fruit	Electrochemical Assay	EQI, µA/V	Reference
Blackberry jam fruit (*Randia formosa* (Jacq.) K. Shum)	SWV	6.78	[34]
Acerola	CV	5.80	[23]
Blackberry (*Rubus* spp.)	DPV	0.738	[21]
Mora de monte (*R. glabratus* Kunth)	DPV	2.26 × 10^−5^	[36]
Açaí	CV	6.9	[23]
Spray-dried açaí powder	DPV	0.168	[21]
Açaí	DPV	1.08	In this work
	CV	2.30	

CV: cyclic voltammetry; DPV: differential pulse voltammetry; SWV: square wave voltammetry.

**Table 3:** Comparative summary of the antioxidant activity methods in existing literature [45].

Method	Mechanism	Sample Suitability	Advantages	Disadvantages	Limitations	Usefulness
DPPH	Electron or H-donation to DPPH radical	Lipophilic	Simple, fast, cost-effective	Not suitable for hydrophilic antioxidants; sensitive to light/pH	Poor correlation to biological systems	Good for rapid screening
ABTS	Electron donation to ABTS^•+^ radical	Hydrophilic and lipophilic	Broad-range applicability, stable	Requires pre-oxidation; possible overestimation	Non-physiological radicals	Excellent for general antioxidant profiling
FRAP	Reduction of Fe^3+^ to Fe^2+^	Hydrophilic	Reproducible, easy	Only reducing power; acidic pH required	Not suitable for all antioxidants	Useful for total reducing capacity
ORAC	Scavenging of peroxyl radicals	Biological fluids, extracts	Physiologically relevant radicals, kinetic data	Time-consuming, costly, fluorescent probe needed	Sensitive to conditions, reproducibility issues	Ideal for biological antioxidant capacity
CUPRAC	Reduction of Cu^2+^ to Cu^+^	Hydrophilic and lipophilic	Stable reagents, broad compatibility	Can have interferences	Not widely used, Cu not biologically relevant	Effective for mixed antioxidant systems
TPC	Reaction with Folin–Ciocalteu reagent	Phenolic compounds	Quick, correlates with phenolic content	Non-specific, including non-phenolics	Not a true antioxidant assay	Useful for phenolic content estimation

**Table 4:** Comparison of EQI with conventional antioxidant assays [45].

Feature	EQI	DPPH	ABTS	FRAP	ORAC	CUPRAC	TPC
Principle	Electron transfer (redox potential and current)	Radical scavenging	Radical scavenging	Reducing power	Peroxyl radical scavenging	Cu^2+^ reducing power	Total reducing compounds
Measurement Type	Electrochemical	Spectrophotometric	Spectrophotometric	Spectrophotometric	Fluorescence	Spectrophotometric	Spectrophotometric
Kinetics	Fast; real-time response	End-point	End-point	End-point	Kinetic	End-point	End-point
Sensitivity	High for electroactive compounds	Moderate	Moderate	Moderate	High	Moderate	Moderate
Selectivity	Selective for electroactive antioxidants	Non-selective	Non-selective	Non-selective	More selective	Non-selective	Poor selectivity
Biological Relevance	Moderate; mimics oxidative mechanisms	Low	Low–Moderate	Low	High	Moderate	Indirect
Cost and Simplicity	Requires potentiostat; moderate training	Simple and cheap	Moderate	Simple	Expensive instrumentation	Moderate	Very simple
Solubility Range	Hydrophilic and lipophilic (depends on electrode material)	Mostly lipophilic	Both	Hydrophilic	Hydrophilic	Both	Hydrophilic
Reproducibility	High, if properly standardized	Variable	Variable	Good	Variable	Good	Variable

## Data Availability

Data will be made available on request.
